# Nutrition survey methods and food composition database update of the Korean Genome and Epidemiology Study

**DOI:** 10.4178/epih.e2024042

**Published:** 2024-04-02

**Authors:** Seon-Joo Park, Jieun Lyu, Kyoungho Lee, Hae-Jeung Lee, Hyun-Young Park

**Affiliations:** 1 Department of Food and Nutrition, College of BioNano Technology, Gachon University, Seongnam, Korea; 2 Institute for Aging and Clinical Nutrition Research, Gachon University, Seongnam, Korea; 3 Division of Population Health Research, Department of Precision Medicine, Korea National Institute of Health, Korea Disease Control and Prevention Agency, Cheongju, Korea; 4 Department of Health Sciences and Technology, Gachon Advanced Institute for Health Science and Technology (GAIHST), Gachon University, Incheon, Korea; 5 Korea National Institute of Health, Korea Disease Control and Prevention Agency, Cheongju, Korea

**Keywords:** Nutrition survey, Database, Korea, Korean Genome and Epidemiology Study

## Abstract

This study presents the nutrition survey methods and the updated food composition database for the Korean Genome and Epidemiology Study (KoGES). The KoGES, which is the largest and longest cohort study in Korea, aims to identify genetic and environmental factors associated with chronic diseases. This study has collected dietary data using a validated semi-quantitative food frequency questionnaire and/or the 24-hour recall method. However, these dietary survey methods use different food composition databases, and their nutritional values are out of date. Therefore, it became necessary to update the food composition database by revising nutrient analysis values to reflect improvements in the performance of food ingredient analysis equipment, revising international values to analysis values of Korean agricultural products, adjusting nutrient units, and adding newly reported nutrients related to chronic diseases. For this purpose, we integrated the different food composition databases used in each nutrition survey, updated 23 nutrients, and expanded 48 new nutrients for 3,648 food items using the latest reliable food composition databases published by national and international institutions. This revised food composition database may help to clarify the relationship between various nutrients and chronic diseases. It could serve as a valuable resource for nutritional, epidemiological, and genomic research and provide a basis for determining public health policies.

## GRAPHICAL ABSTRACT

[Fig f3-epih-46-e2024042]

## Key Message

The Korean Genome and Epidemiology Study (KoGES) is the largest and longest-running cohort study in Korea aimed at identifying the relationship between chronic diseases and nutrient intake. This paper provides a detailed methods of the nutritional surveys and introduces updates to the existing 23 nutrients and the addition of 48 new nutrients. These enhancements are expected to significantly increase the utility of the KoGES nutritional data.

## INTRODUCTION

The Korea Genome and Epidemiology Study (KoGES) is the largest cohort study in Korea, and it has been conducted by the Korea Disease Control and Prevention Agency since 2001. The purpose of the KoGES is to identify the associations between genetic and environmental factors and interactions with chronic diseases prevalent among Koreans. The general profile of the KoGES has been described previously [1]. However, a detailed description of the dietary survey methods and food composition databases used to calculate nutrient intake has not yet been published. The nutrition survey of the KoGES has been conducted using a semiquantitative food frequency questionnaire (FFQ) and/or 24-hour recall methods according to the characteristics of the sub-cohort. The nutrient intake of the FFQ was calculated using the food composition table of the Korean Nutrition Society published in 2000 [2], and the nutrient intakes for 24-hour recall were calculated by modifying the food composition table of the Rural Development Administration, published in 2006 [3]. These dietary survey methods use different food composition databases; furthermore, their nutritional values are outdated and need integration and updating to reflect current knowledge regarding the nutritional content of foods. Therefore, it is necessary to revise the food composition database by addressing inaccuracies in nutrient analysis values resulting from advancements in food ingredient analysis technology, revising overseas values to analysis values of Korean agricultural products, adjusting nutrient units, and adding nutrients that have been newly reported to be related to chronic diseases.

Food composition databases play a pivotal role in estimating food and nutrient intake at individual and population levels, which is important for elucidating the relationship between nutrient intake and diseases [4]. Regular database updates, such as expanding the number of nutrients reported to be related to chronic diseases, are imperative for improving quality and meeting the evolving needs of data users.

Recent international studies have reported relationships between the intake of various nutrients, such as dietary fiber [5], branched-chain amino acids [6], sugar [7], and saturated fatty acids [8], and chronic diseases. However, these nutrients are not currently released by KoGES.

The Korean food composition table, published by the Rural Development Administration, is a representative food composition table in Korea that displays the nutrients per 100 g of food. In 2017, the Korea Food and Drug Administration expanded the number of nutrients to 130 and increased the number of foods analyzed in Korea [9,10]. These updates need to be reflected in the KoGES food composition database.

In this study, we (1) present detailed information on the nutrition survey of the KoGES, (2) describe the process of revising the food composition database, (3) present the changes in the revision of the food composition database using previously collected dietary survey data and discuss the reasons for the changes, and (4) suggest considerations for using the revised food composition database.

## NUTRITION SURVEY METHODS OF THE KOREA GENOME AND EPIDEMIOLOGY STUDY

### Nutrition survey tools of the Korea Genome and Epidemiology Study

The KoGES uses a validated FFQ and the 24-hour recall method to assess the nutritional status of the participants. A 103-item FFQ was developed for the KoGES dietary assessment in 2001 [11] and then expanded to 106 items in 2004 to investigate participants’ eating habits more accurately. In the 106-item FFQ, the number of cooked rice types increased from 3 to 5, and meat-based soups (*tang*) (e.g., *sullungtang, gomtang*, etc.), which are frequently consumed by Koreans, were added. The baseline survey (2001-2002) in the Ansan and Ansung cohort study used a 103-item FFQ.

In the FFQ questionnaire, the frequency of food intake was classified into 9 categories. Portion size was categorized as small, medium, or large. For seasonal fruits and vegetables, participants reported their intake, frequency, and amount at 3 months, 6 months, 9 months, and 12 months, respectively. To ensure the accuracy of the survey, representative pictures of large, medium, and small portions of food were included in the questionnaire to help participants (Supplementary Material 1). Nutrient intake from each food item in the FFQ was calculated using weights derived from the consumption frequency and portion size of each food item. The daily nutrient intake of each individual was calculated as the sum of the nutrient intake from each food item. The KoGES used the FFQ as the main dietary survey intake method, with 24-hour recall used as an additional method for dietary measurement. The Health Examinee (HEXA) study collected 2 non-consecutive days of 24-hour recall data. The first-day survey was conducted face-to-face at the examination center, while the second-day survey was conducted via telephone interviews. The immigrant study collected 1-day 24-hour recall data via face-to-face interviews.

### Protocol of the Korea Genome and Epidemiology Study nutrition survey

**Figure 1.**
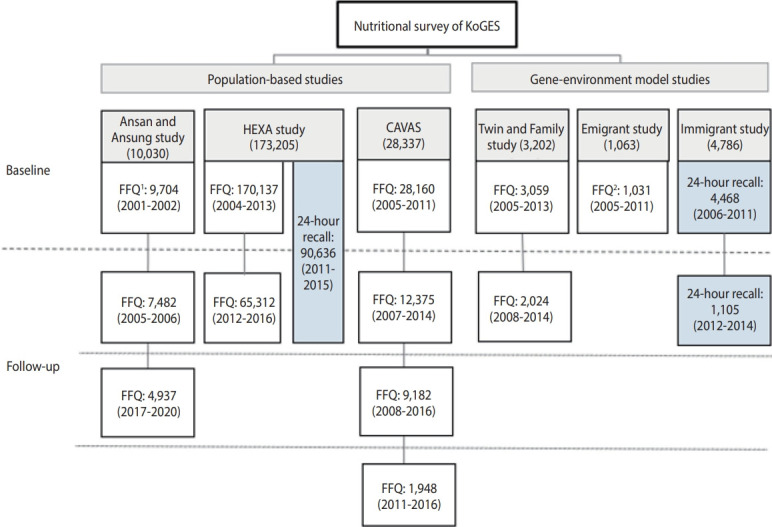
Nutrition survey period, tools, and participants of KoGES (February 2023). KoGES, Korean Genome and Epidemiology Study; HEXA, Health Examinee; CAVAS, Cardiovascular Disease Association Study; FFQ, food frequency questionnaire. 1103 Food items FFQ. 2Self-developed FFQ.

Figure 1 presents the protocol of the nutrition survey, including the dietary assessment tool, duration of the nutrition survey, and number of participants in each sub-cohort.

In the Ansan and Ansung study, 103-item FFQ data were collected from 9,704 participants during the baseline survey (2001-2002). In 2 follow-up surveys conducted in 2005-2006 and 2017-2020, 106-item FFQ data were collected from 7,482 and 4,937 participants, respectively. In the HEXA study, 106-item FFQ data were collected from 170,137 participants during the baseline survey and 65,312 participants during the follow-up survey. Additionally, 2 days of non-consecutive 24-hour recall data were collected from 90,636 participants between 2011 and 2015. The Cardiovascular Disease Association Study (CAVAS) collected FFQ data from 28,160 individuals during a baseline survey (2005-2011) and follow-up FFQ data during 2007-2016. The Twin and Family study collected FFQ data from 3,059 participants in the baseline survey (2005-2013) and 2,024 participants in the follow-up study (2008-2014). For the Japanese Emigrant study, FFQ data were collected from 1,031 baseline survey participants using a self-developed FFQ to reflect the eating habits of Korean emigrants living in Japan. In the Immigrant study, the nutrient intake of Vietnamese women and their Korean husbands was assessed using the 24-hour recall method, with 4,468 participants in the baseline survey and 1,105 participants in the follow-up survey.

## REVISION PROCESS OF THE FOOD COMPOSITION DATABASE

**Figure 2.**
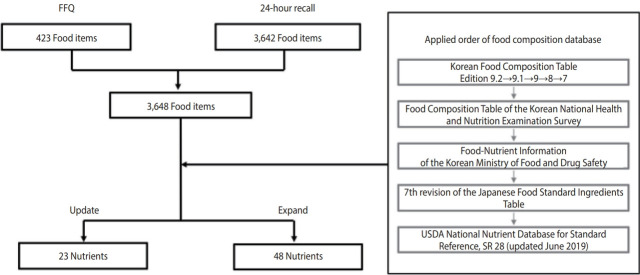
Revision process of food composition database. FFQ, food frequency questionnaire; USDA, United States Department of Agriculture.

The KoGES currently releases information on the intake of 23 nutrients: energy, protein, fat, carbohydrates, ash, fiber, calcium, phosphorus, iron, potassium, zinc, sodium, vitamin A, retinol, β-carotene, vitamin E, vitamin B_1_, vitamin B_2_, niacin, vitamin C, vitamin B_6_, folate, and cholesterol. The process of revising the food composition database is shown in Figure 2.

### Integrating the food composition databases of both dietary assessment methods

To integrate the food composition database used for the FFQ with that used for the 24-hour recall method, the 423 foods used in the FFQ were matched to foods in the food composition database used for 24-hour recall. First, Korean and English food names were matched. Then, the energy, carbohydrate, protein, fat, and water content of each food were compared. If the nutrient differences were significant (≥ ± 20%), even when the Korean name or English name was the same, other similar foods with similar nutrient contents were matched (for example, “Chicken, Meat and skin, Stewed” matched with “Chicken, Meat, Breast, Stewed”). An expert reviewed the suitability of the matched foods. The final target food list for update and expansion included 3,648 foods, consisting of 423 food items used in the FFQ and 3,642 food items used in the 24-hour recall method.

### Selection of food composition databases and revision process

In this study, an indirect method was applied using the latest and most reliable food composition databases published by national institutions. We assessed the availability of Korean and international food composition databases and selected 7 Korean and 2 international databases, as follows: Korean Food Composition Table edition 9.2 [12] and Korean Food Composition Table edition 9.1 [10]. Korean Food Composition Table, 9th edition [13], Korean Food Composition Table, 8th edition [14]. Korean Food Composition Table, 7th edition [3], Food-Nutrient Information of the Korean National Health and Nutrition Examination Survey [15], Food Nutrient Database of the Korean Ministry of Food and Drug Safety [16], the 7th revision of the Japanese Food Standard Ingredients Table [17], and United States Department of Agriculture (USDA) National Nutrient Database for Standard Reference, SR 28 (updated in June 2019) [18].

The process of matching the 3,648 foods with each food composition database was as follows: First, Korean food names, English food names, and food conditions (raw, cooked, dry, etc.) were considered. Second, the energy, carbohydrate, protein, fat, and water content of each food were compared. If the nutrient differences were significant (≥ ± 20%), even when they had the same names, the food with the most similar cooking or processing status was substituted. If there were no similar foods, they were matched by considering the following factors: cooking, processing, drying conditions, and moisture content. For processed food with a specific brand product name, the energy and fat values were assessed using information on the company webpage and matched.

As for the order of application, if a nutrient was found in the most recently published food composition table, that value was applied. The food composition table values were reviewed sequentially, and if there were no newly published nutrient values, existing nutrient values were used. The sequence of applying the food composition databases was as follows: Korean Food Composition Table edition 9.2 [12], Korean Food Composition Table edition 9.1 [10], Korean Food Composition Table, 9th edition [13], Korean Food Composition Table, 8th edition [14], Korean Food Composition Table, 7th edition [3], Food Composition Table of the Korean National Health and Nutrition Examination Survey [15], Food Nutrient Database of the Korean Ministry of Food and Drug Safety [16], 7th revision of the Japanese Food Standard Ingredients Table [17], and the USDA National Nutrient Database for Standard Reference, SR 28 (updated in June 2019) [18].

### Units of nutrients

Vitamin B6 values in the previous food composition database used pyridoxine values. The most recently published food composition database had a total vitamin B_6_ value; however, the number of foods with total vitamin B_6_ values was too small to use. Therefore, we updated the vitamin B_6_ values using the pyridoxine values. Previously, fiber intake was calculated using crude fiber data; however, with this revision, fiber values were updated with the total dietary fiber value.

The pre-revision unit for vitamin A was the retinol equivalent (RE). However, since 2015, the Dietary Reference Intakes for Koreans have used retinol activity equivalent (RAE) as the unit of vitamin A. Therefore, both RE and RAE were presented in the revised food composition database.

RE and RAE were calculated using the following formulas based on retinol and carotene contents.


RE (retinol equivalent)= retinol+(β-carotene/6)

RAE (retinol activity equivalent)= retinol+(β-carotene/12)

### Comparison of nutrient intake between pre- and post-revision

To observe the differences resulting from the revision of the food composition database, changes in the intake of 23 nutrients before and after the revision were compared using the food frequency questionnaire data of 205,785 participants (170,143 in the HEXA study; 28,160 in the CAVAS; and 7,482 in the Ansan and Ansung study). We calculated the absolute and percentage changes in 23 nutrients. The mean and standard deviation of each nutrient were calculated, and the paired t-test was performed to compare the pre-revision and post-revision values.

The percentage difference between the pre-revision and post-revision values was calculated as follows:


Change (%)= [(Post-revision value−Pre-revision value)/Pre-revision value]× 100

All nutrient intake calculations and statistical analyses were performed using SAS version 9.4 (SAS Institute Inc., Cary, NC, USA).

### Changes in nutrient intake in the revision of the food composition database and reasons for the changes

Table 1 shows the changes in nutrient intake before and after updating the food composition database for the 205,785 participants. The energy and carbohydrate intake increased by 51.2 kcal (3.1%) and 16.3 g (5.4%), respectively, while protein and fat intake decreased by 1.6 g (-2.5%) and 1.4 g (-6.6%), respectively (Table 1).

Sodium intake (from 2,575.2 to 1,977.7 mg, -22.2%) decreased due to the decrease in sodium of cabbage kimchi, a major source of sodium in Korea, from 1,146.0 mg to 624.0 mg per 100 g (-522.0 mg). The nutrient that increased the most after the update was fiber (348.4%). Among the 423 foods used in the FFQ, the foods with the largest change in fiber content per 100 g and the highest fiber contribution were cooked white rice (0.1-1.2 g) and black beans (4.0-20.8 g). The nutrient with the largest decrease was vitamin B_6_ (55.2%). This was due to a decrease in the amount of vitamin B_6_ (pyridoxine) per 100 g of white sugar (0.9-0.0 g), bean sprouts (0.96-0.01 g), black beans (0.54-0.05 g), and cooked white rice (0.06-0.02 g) (data not shown).

These changes in nutrient intake were caused by changes in the nutrient content of the same foods. The differences in the nutrient content for each food may be attributed to improved analytical methods and techniques, better food sampling methods, and the replacement of international food composition table values with Korean analytical values [19,20]. As shown by these results, changes in database values for frequently consumed foods can significantly affect an individual’s nutrient intake [20].

Changes in the participants’ intake of 23 nutrients across the 3 subcohorts (Ansan and Ansung study, HEXA study, and CAVAS) are presented in Supplementary Material 2.

### Selection of expanded nutrients

To select an expanded range of nutrients, we conducted an online survey to examine the needs of KoGES data users and academic society members and a literature review to select nutrients that recent publications have identified as relevant to chronic diseases. From an expert consultation meeting to discuss the results of the online survey and literature review, 48 nutrients were selected that were highly recommended by researchers and correlated with chronic diseases (Table 2). The 48 selected nutrients are as follows: total sugar, soluble fiber, insoluble fiber, vitamin D, vitamin B_12_, magnesium, iodine, selenium, copper, branched-chain amino acids (histidine, leucine, isoleucine, lysine, valine, phenylalanine, threonine, tryptophan, methionine), and fatty acids (saturated fatty acid, monounsaturated fatty acid, polyunsaturated fatty acid, omega-3 fatty acid, omega-6 fatty acid, capric acid, lauric acid, myristic acid, palmitic acid, stearic acid, arachidic acid, behenic acid, lignoceric acid, myristoleic acid, palmitoleic acid, oleic acid, gadoleic acid, erucic acid, nervonic acid, linoleic acid, α-linolenic acid, γ-linolenic acid, stearidonic acid, eicosadienic acid, icosatrienoic acid, arachidonic acid, eicosapentaenoic acid, docosadienoic acid, docosapentaenoic acid, and docosahexaenoic acid).

The new 48 nutrient values for the 3,648 foods were constructed using the same process as the 23 nutrient updates. Table 3 shows the newly expanded nutrient intakes for 205,785 participants. The coverage of the 423 foods with the 48 newly expanded nutrients is shown in Supplementary Material 3. The coverage of the 423 foods used in the FFQ ranged from 58.6% (soluble/insoluble fiber) to 99.5% (vitamin B_12_). Therefore, when researchers use these data to analyze the relationship between the intake of soluble and insoluble dietary fiber, vitamin D, magnesium, copper, iodine, and amino acids and disease, they should consider the possibility that the intake has been underestimated.

## CONCLUSION

In this study, we introduced the nutritional survey methods, the current status of the survey and results of the updated KoGES food composition database. For a total of 3,648 foods, 23 nutrients were updated and 48 nutrients were newly included using Korean and international nutrient food composition databases. The updated food composition database described in this study reflects the most recently published food composition table from reliable national institutes; thus, using this database is likely to improve the accuracy of research on the dietary intake of Koreans. In addition, the expanded range of nutrients may be useful for researchers aiming to analyze the relationships between various nutrients and chronic diseases.

### Ethics statement

This study was approved by the Institutional Review Boards of Korea Disease Control and Prevention Agency (KDCA) (IRB No. 2018-03-05-P-A).

## Figures and Tables

**Figure f3-epih-46-e2024042:**
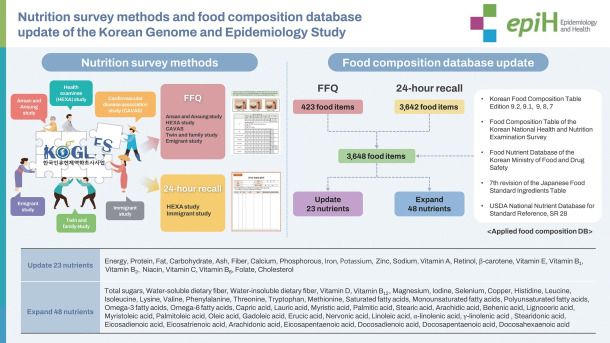


**Table 1.** Comparison of nutrient intakes after revision of the food composition database for 3 pooled cohorts (n=205,785)^1^

Nutrients	Pre-revision value	Post-revision value	Overall difference	% of pre-revision value	(Post-revision value/Pre-revision value)×100 (%)	Change (%) [(Post-revision value–Pre-revision value)/Pre-revision value]×100	p-value^2^
Energy (kcal)	1,746.40±1.30	1,797.60±1.32	51.2	1.0	103.1	3.1	<0.001
Protein (g)	58.80±0.06	57.20±0.06	-1.6	1.0	97.5	-2.5	<0.001
Fat (g)	27.40±0.04	26.00±0.04	-1.4	0.9	93.4	-6.6	<0.001
Carbohydrate (g)	311.80±0.21	328.00±0.22	16.3	1.1	105.4	5.4	<0.001
Ash (g)	15.70±0.02	10.10±0.01	-5.5	0.7	68.4	-31.6	<0.001
Fiber (g)	5.80±0.01	24.50±0.02	18.7	4.5	448.4	348.4	<0.001
Calcium (mg)	442.70±0.61	465.10±0.61	22.5	1.1	107.2	7.2	<0.001
Phosphorous (mg)	888.20±0.84	800.30±0.84	-87.9	0.9	88.8	-11.2	<0.001
Iron (mg)	9.90±0.01	8.90±0.01	-1.1	0.9	93.0	-7.0	<0.001
Potassium (mg)	2,245.60±2.48	1,972.80±2.30	-272.8	0.9	87.5	-12.5	<0.001
Zinc (mg)	7.90±0.01	8.20±0.01	0.3	1.0	104.8	4.8	<0.001
Sodium (mg)	2,575.20±3.35	1,977.70±2.66	-597.5	0.8	77.8	-22.2	<0.001
Vitamin A (RE)	479.60±0.81	487.20±0.85	7.6	1.0	103.1	3.1	<0.001
Retinol (µg)	66.70±0.14	91.80±0.20	25.2	1.3	133.5	33.5	<0.001
β-carotene (µg)	2,404.80±4.40	2,373.20±4.56	-31.6	1.0	99.9	-0.1	<0.001
Vitamin E (mg)	8.10±0.01	7.70±0.01	-0.4	1.0	97.0	-3.0	<0.001
Vitamin B_1_ (mg)	1.00±0.00	0.80±0.00	-0.2	0.8	83.6	-16.4	<0.001
Vitamin B_2_ (mg)	0.90±0.00	1.10±0.00	0.2	1.2	116.4	16.4	<0.001
Niacin (mg)	14.30±0.01	9.70±0.01	-4.6	0.7	66.2	-33.8	<0.001
Vitamin C (mg)	106.30±0.16	71.00±0.11	-35.4	0.7	66.8	-33.2	<0.001
Vitamin B_6_ (mg)	1.60±0.00	0.70±0.00	-0.9	0.4	44.8	-55.2	<0.001
Folate (μg)	217.50±0.29	237.10±0.28	19.5	1.1	113.4	13.4	<0.001
Cholesterol (mg)	161.40±0.29	138.20±0.24	-23.3	0.9	88.6	-11.4	<0.001

Values are presented as mean±standard error. RE, retinol equivalent.

1 Three pooled cohorts: Ansan and Ansung study, Health Examinee (HEXA) study, Cardiovascular Disease Association Study (CAVAS).

2 Paired t-test.

**Table 2.** Expanded nutrient names and reasons for selection

Category (n)	Nutrients	Reason for selection
Dietary fiber (2)	Soluble	Diabetes, obesity, cardiovascular disease
	Insoluble	
Vitamins (2)	Vitamin D	High demand for researchers. diabetes, metabolic syndrome, hypertension, obesity
	Vitamin B_12_	Diabetes, hypertension, obesity
Minerals (4)	Magnesium	Diabetes, hypertension, cardiovascular disease
	Iodine	Diabetes, hypertension, obesity
	Selenium	Diabetes, hypertension
	Copper	Diabetes, metabolic syndrome, cardiovascular disease
Branched-chain amino acids (9)	Histidine, leucine, isoleucine, lysine, valine, phenylalanine, threonine, tryptophan, methionine	Diabetes, hyperlipidemia
Sugar	Total sugar	Diabetes, metabolic syndrome, hypertension, obesity, hyperlipidemia, cardiovascular disease
Fatty acids (30)	Saturated fatty acids, monounsaturated fatty acids, polyunsaturated fatty acids, omega-3 fatty acids, omega-6 fatty acids, capric acid, lauric acid, myristic acid, palmitic acid, stearic acid, arachidic acid, behenic acid, lignoceric acid, myristoleic acid, palmitoleic acid, oleic acid, gadoleic acid, erucic acid, nervonic acid, linoleic acid, α-linolenic acid, γ-linolenic acid, stearidonic acid, eicosadienic acid, eicosatrienoic acid, arachidonic acid, eicosapentaenoic acid, docosadienoic acid, docosapentaenoic acid, docosahexaenoic acid	High demand for researchers, diabetes, metabolic syndrome, obesity, hyperlipidemia, cardiovascular disease

**Table 3.** Intake of 48 expanded nutrients in KoGES participants (n=205,785)

Nutrients	Mean±SE
Total sugars (g)	52.90±0.07
Water-soluble dietary fiber (g)	3.50±0.01
Water-insoluble dietary fiber (g)	9.30±0.01
Vitamin D (μg)	7.70±0.01
Vitamin B_12_ (μg)	4.80±0.01
Magnesium (mg)	139.20±0.19
Iodine (μg)	336.50±1.05
Selenium (μg)	38.60±0.06
Copper (mg)	0.40±0.00
Histidine (mg)	728.90±1.08
Leucine (mg)	1,976.20±2.89
Isoleucine (mg)	1,017.40±1.49
Lysine (mg)	1,534.90±2.32
Valine (mg)	1,218.30±1.78
Phenylalanine (mg)	1,171.90±1.69
Threonine (mg)	1,085.00±1.58
Tryptophan (mg)	284.80±0.41
Methionine (mg)	530.50±0.80
Saturated fatty acids (g)	9.90±0.02
Monounsaturated fatty acids (g)	8.10±0.01
Polyunsaturated fatty acids (g)	5.20±0.01
Omega-3 fatty acids (g)	1.00±0.00
Omega-6 fatty acids (g)	4.10±0.01
Capric acid (mg)	162.40±0.30
Lauric acid (mg)	702.70±1.39
Myristic acid (mg)	804.30±1.37
Palmitic acid (mg)	4,980.80±8.35
Stearic acid (mg)	1,972.50±3.59
Arachidic acid (mg)	54.40±0.09
Behenic acid (mg)	33.30±0.08
Lignoceric acid (mg)	22.50±0.04
Myristoleic acid (mg)	58.30±0.17
Palmitoleic acid (mg)	394.90±0.77
Oleic acid (mg)	6,230.10±11.02
Gadoleic acid (mg)	133.80±0.26
Erucic acid (mg)	47.10±0.13
Nervonic acid (mg)	6.00±0.01
Linoleic acid (mg)	3,814.90±5.96
α-linolenic acid (mg)	517.00±0.81
γ-linolenic acid (mg)	7.30±0.02
Stearidonic acid (mg)	21.60±0.06
Eicosadienoic acid (mg)	42.40±0.10
Eicosatrienoic acid (mg)	16.70±0.03
Arachidonic acid (mg)	44.00±0.12
Eicosapentaenoic acid (mg)	130.90±0.30
Docosadienoic acid (mg)	3.30±0.01
Docosapentaenoic acid (mg)	29.40±0.07
Docosahexaenoic acid (mg)	249.00±0.56

KoGES, Korean Genome and Epidemiology Study; SE, standard error.

## Supplementary materials

Supplementary Material 1. Food frequency questionnaire of KoGES

Supplementary Material 2. Comparison of nutrient intake after revision of the food composition database in the three subcohorts

Supplementary Material 3. Coverage status of newly expanded nutrients
