# Supplementary figures and images for: Nutrition survey methods and food composition database update of the Korean Genome and Epidemiology Study
Source: Epidemiol Health. 2024 Apr 2;46:e2024042. doi: 10.4178/epih.e2024042 (PMC11417449; doi:10.4178/epih.e2024042)

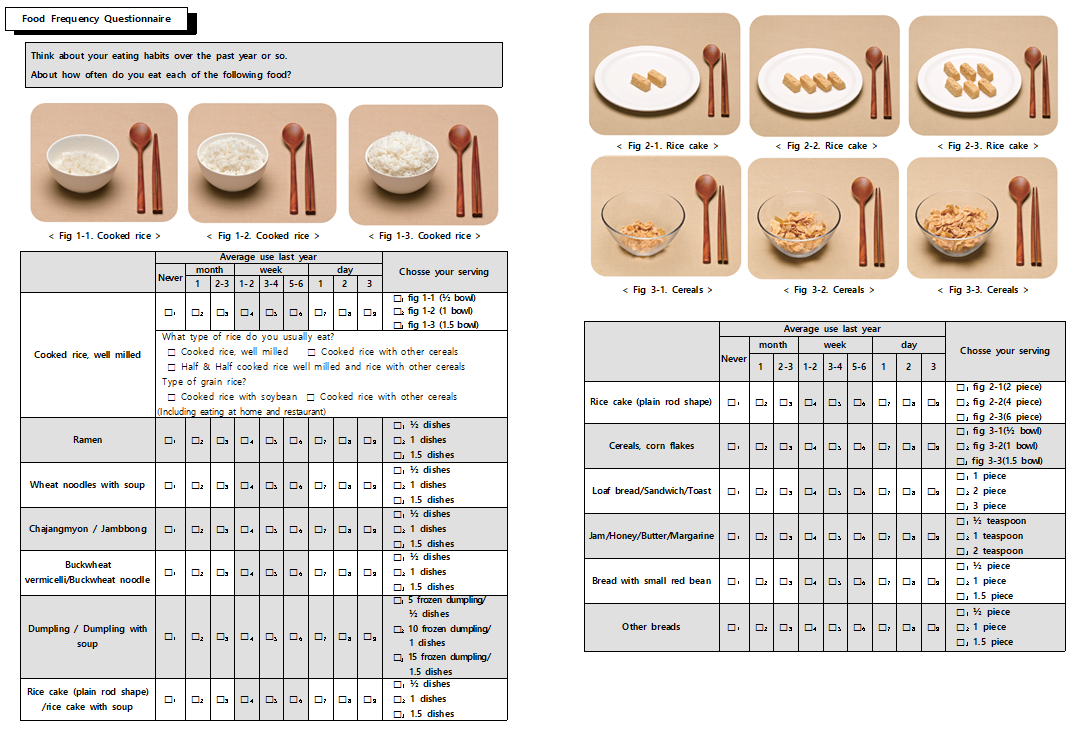


**Supplementary Material 1.** Food frequency questionnaire of KoGES

Supplement: Supplementary Material 1. — Food frequency questionnaire of KoGES [file epih-46-e2024042-Supplementary-1.docx]
