# Supplementary material for: Nutrition survey methods and food composition database update of the Korean Genome and Epidemiology Study
Source: Epidemiol Health. 2024 Apr 2;46:e2024042. doi: 10.4178/epih.e2024042 (PMC11417449; doi:10.4178/epih.e2024042)
Supplement: Supplementary Material 2. — Comparison of nutrient intake after revision of the food composition database in the three subcohorts [file epih-46-e2024042-Supplementary-2.docx]

**Supplementary Material 2.** Comparison of nutrient intake after revision of the food composition database in the three subcohorts

| Nutrients ^1^ | Values pre-revision | | | Values post-revision | | | Overall difference | % to values pre-revision | Values post-revision/ Values pre-revision x 100 (%) | Change (%)  [(Values post-revision – Values pre-revision) /  Values pre-revision] × 100 | *P* value ^2^ |
| --- | --- | --- | --- | --- | --- | --- | --- | --- | --- | --- | --- |
| Ansan and Ansung study (N=7482) | | | | | | | | | | | |
| Energy (kcal) | 1784.02 | ± | 7.10 | 1840.25 | ± | 7.11 | 56.2 | 1.0 | 103.4 | 3.4 | <0.0001 |
| Protein (g) | 58.51 | ± | 0.32 | 56.95 | ± | 0.30 | -1.6 | 1.0 | 97.7 | -2.3 | <0.0001 |
| Fat (g) | 27.70 | ± | 0.23 | 26.35 | ± | 0.24 | -1.3 | 0.9 | 93.4 | -6.6 | <0.0001 |
| Carbohydrate (g) | 320.11 | ± | 1.13 | 337.19 | ± | 1.15 | 17.1 | 1.1 | 105.6 | 5.6 | <0.0001 |
| Ash (g) | 429.98 | ± | 3.08 | 10.39 | ± | 0.07 | -6.6 | 0.7 | 65.7 | -34.3 | <0.0001 |
| Fiber (g) | 892.09 | ± | 4.36 | 25.26 | ± | 0.11 | 19.3 | 4.5 | 453.1 | 353.1 | <0.0001 |
| Calcium (mg) | 9.71 | ± | 0.06 | 456.66 | ± | 3.10 | 26.7 | 1.1 | 108.4 | 8.4 | <0.0001 |
| Phosphorous (mg) | 2272.63 | ± | 12.74 | 793.44 | ± | 4.37 | -98.6 | 0.9 | 87.6 | -12.4 | <0.0001 |
| Iron (mg) | 474.22 | ± | 4.26 | 8.89 | ± | 0.04 | -0.8 | 1.0 | 95.2 | -4.8 | <0.0001 |
| Potassium (mg) | 2733.40 | ± | 18.44 | 1978.54 | ± | 11.36 | -294.1 | 0.9 | 87.4 | -12.6 | <0.0001 |
| Zinc (mg) | 1.02 | ± | 0.01 | 8.09 | ± | 0.04 | 0.4 | 1.1 | 105.1 | 5.1 | <0.0001 |
| Sodium (mg) | 0.89 | ± | 0.01 | 2085.06 | ± | 14.62 | -648.3 | 0.8 | 77.2 | -22.8 | <0.0001 |
| Vitamin A (RE) | 13.92 | ± | 0.08 | 457.42 | ± | 4.18 | -16.8 | 1.0 | 98.6 | -1.4 | <0.0001 |
| Retinol (µg) | 102.44 | ± | 0.75 | 82.98 | ± | 1.01 | 24.0 | 1.4 | 136.3 | 36.3 | <0.0001 |
| β-carotene (µg) | 7.73 | ± | 0.04 | 2247.73 | ± | 22.53 | -164.0 | 1.0 | 95.4 | -4.6 | <0.0001 |
| Vitamin E (mg) | 1.58 | ± | 0.01 | 8.36 | ± | 0.06 | 0.3 | 1.1 | 108.9 | 8.9 | <0.0001 |
| Vitamin B_1_ (mg) | 218.61 | ± | 1.48 | 0.82 | ± | 0.01 | -0.2 | 0.8 | 80.4 | -19.6 | <0.0001 |
| Vitamin B_2_ (mg) | 58.97 | ± | 0.68 | 1.06 | ± | 0.01 | 0.2 | 1.2 | 118.4 | 18.4 | <0.0001 |
| Niacin (mg) | 2411.75 | ± | 23.24 | 9.15 | ± | 0.06 | -4.8 | 0.6 | 64.5 | -35.5 | <0.0001 |
| Vitamin C (mg) | 16.98 | ± | 0.14 | 64.95 | ± | 0.51 | -37.5 | 0.6 | 63.7 | -36.3 | <0.0001 |
| Vitamin B_6_ (mg) | 5.91 | ± | 0.03 | 0.70 | ± | 0.00 | -0.9 | 0.4 | 44.6 | -55.4 | <0.0001 |
| Folate (μg) | 8.03 | ± | 0.06 | 238.90 | ± | 1.37 | 20.3 | 1.2 | 115.1 | 15.1 | <0.0001 |
| Cholesterol (mg) | 148.22 | ± | 1.52 | 127.93 | ± | 1.29 | -20.3 | 1.1 | 112.5 | 12.5 | <0.0001 |
|  |  |  |  |  |  |  |  |  |  |  |  |
| HEXA study (N=170,143) | | | | | | | | | | | |
| Energy (kcal) | 1759.24 | ± | 1.44 | 1809.91 | ± | 1.45 | 50.7 | 1.0 | 103.0 | 3.0 | <0.0001 |
| Protein (g) | 59.91 | ± | 0.07 | 58.23 | ± | 0.06 | -1.7 | 1.0 | 97.4 | -2.6 | <0.0001 |
| Fat (g) | 28.21 | ± | 0.05 | 26.79 | ± | 0.05 | -1.4 | 0.9 | 93.8 | -6.2 | <0.0001 |
| Carbohydrate (g) | 312.28 | ± | 0.23 | 328.52 | ± | 0.24 | 16.2 | 1.1 | 105.4 | 5.4 | <0.0001 |
| Ash (g) | 452.51 | ± | 0.67 | 10.24 | ± | 0.01 | -5.3 | 0.7 | 69.2 | -30.8 | <0.0001 |
| Fiber (g) | 901.19 | ± | 0.92 | 24.65 | ± | 0.03 | 18.8 | 4.5 | 446.5 | 346.5 | <0.0001 |
| Calcium (mg) | 10.16 | ± | 0.01 | 474.73 | ± | 0.68 | 22.2 | 1.1 | 106.8 | 6.8 | <0.0001 |
| Phosphorous (mg) | 2280.55 | ± | 2.72 | 816.51 | ± | 0.92 | -84.7 | 0.9 | 89.4 | -10.6 | <0.0001 |
| Iron (mg) | 490.59 | ± | 0.90 | 8.98 | ± | 0.01 | -1.2 | 0.9 | 91.7 | -8.3 | <0.0001 |
| Potassium (mg) | 2559.99 | ± | 3.63 | 2008.87 | ± | 2.54 | -271.7 | 0.9 | 87.8 | -12.2 | <0.0001 |
| Zinc (mg) | 1.02 | ± | 0.00 | 8.39 | ± | 0.01 | 0.3 | 1.0 | 104.7 | 4.7 | <0.0001 |
| Sodium (mg) | 0.92 | ± | 0.00 | 1971.71 | ± | 2.88 | -588.3 | 0.8 | 78.1 | -21.9 | <0.0001 |
| Vitamin A (RE) | 14.61 | ± | 0.02 | 503.07 | ± | 0.94 | 12.5 | 1.0 | 103.9 | 3.9 | <0.0001 |
| Retinol (µg) | 108.45 | ± | 0.18 | 96.24 | ± | 0.23 | 26.2 | 1.3 | 133.4 | 33.4 | <0.0001 |
| β-carotene (µg) | 8.05 | ± | 0.01 | 2442.00 | ± | 5.08 | -7.5 | 1.0 | 100.7 | 0.7 | <0.0001 |
| Vitamin E (mg) | 1.61 | ± | 0.00 | 7.81 | ± | 0.01 | -0.5 | 1.0 | 96.3 | -3.7 | <0.0001 |
| Vitamin B_1_ (mg) | 221.31 | ± | 0.31 | 0.86 | ± | 0.00 | -0.2 | 0.8 | 84.1 | -15.9 | <0.0001 |
| Vitamin B_2_ (mg) | 70.04 | ± | 0.16 | 1.08 | ± | 0.00 | 0.2 | 1.2 | 116.4 | 16.4 | <0.0001 |
| Niacin (mg) | 2449.52 | ± | 4.83 | 9.98 | ± | 0.01 | -4.6 | 0.7 | 67.0 | -33.0 | <0.0001 |
| Vitamin C (mg) | 15.54 | ± | 0.03 | 72.92 | ± | 0.13 | -35.5 | 0.7 | 67.3 | -32.7 | <0.0001 |
| Vitamin B_6_ (mg) | 5.85 | ± | 0.01 | 0.71 | ± | 0.00 | -0.9 | 0.4 | 44.7 | -55.3 | <0.0001 |
| Folate (μg) | 8.28 | ± | 0.01 | 241.24 | ± | 0.31 | 19.9 | 1.1 | 113.2 | 13.2 | <0.0001 |
| Cholesterol (mg) | 167.73 | ± | 0.32 | 143.48 | ± | 0.26 | -24.2 | 0.9 | 87.6 | -12.4 | <0.0001 |
|  |  |  |  |  |  |  |  |  |  |  |  |
| CAVAS (N=28,160) | | | | | | | | | | | |
| Energy (kcal) | 1658.89 | ± | 3.43 | 1712.10 | ± | 3.46 | 53.2 | 1.0 | 103.4 | 3.4 | <0.0001 |
| Protein (g) | 52.28 | ± | 0.16 | 51.05 | ± | 0.15 | -1.2 | 1.0 | 98.1 | -1.9 | <0.0001 |
| Fat (g) | 22.28 | ± | 0.11 | 20.93 | ± | 0.11 | -1.4 | 0.9 | 91.4 | -8.6 | <0.0001 |
| Carbohydrate (g) | 306.61 | ± | 0.56 | 322.78 | ± | 0.57 | 16.2 | 1.1 | 105.4 | 5.4 | <0.0001 |
| Ash (g) | 386.47 | ± | 1.55 | 9.47 | ± | 0.03 | -6.7 | 0.6 | 64.1 | -35.9 | <0.0001 |
| Fiber (g) | 808.63 | ± | 2.17 | 23.29 | ± | 0.06 | 17.8 | 4.6 | 458.3 | 358.3 | <0.0001 |
| Calcium (mg) | 8.63 | ± | 0.03 | 409.46 | ± | 1.55 | 23.0 | 1.1 | 108.9 | 8.9 | <0.0001 |
| Phosphorous (mg) | 2027.08 | ± | 6.59 | 704.37 | ± | 2.16 | -104.3 | 0.9 | 85.4 | -14.6 | <0.0001 |
| Iron (mg) | 414.90 | ± | 2.16 | 8.18 | ± | 0.02 | -0.5 | 1.0 | 100.0 | 0.0 | <0.0001 |
| Potassium (mg) | 2624.75 | ± | 9.78 | 1753.26 | ± | 5.99 | -273.8 | 0.9 | 86.2 | -13.8 | <0.0001 |
| Zinc (mg) | 0.91 | ± | 0.00 | 7.36 | ± | 0.02 | 0.3 | 1.1 | 105.2 | 5.2 | <0.0001 |
| Sodium (mg) | 0.77 | ± | 0.00 | 1984.95 | ± | 7.73 | -639.8 | 0.8 | 76.2 | -23.8 | <0.0001 |
| Vitamin A (RE) | 12.69 | ± | 0.04 | 399.34 | ± | 2.10 | -15.6 | 1.0 | 99.2 | -0.8 | <0.0001 |
| Retinol (µg) | 94.61 | ± | 0.42 | 67.63 | ± | 0.49 | 19.1 | 1.3 | 133.3 | 33.3 | <0.0001 |
| β-carotene (µg) | 7.03 | ± | 0.02 | 1990.88 | ± | 11.30 | -141.7 | 1.0 | 96.4 | -3.6 | <0.0001 |
| Vitamin E (mg) | 1.44 | ± | 0.00 | 6.77 | ± | 0.03 | -0.3 | 1.0 | 98.0 | -2.0 | <0.0001 |
| Vitamin B_1_ (mg) | 194.48 | ± | 0.75 | 0.75 | ± | 0.00 | -0.2 | 0.8 | 81.8 | -18.2 | <0.0001 |
| Vitamin B_2_ (mg) | 48.51 | ± | 0.34 | 0.90 | ± | 0.00 | 0.1 | 1.2 | 115.6 | 15.6 | <0.0001 |
| Niacin (mg) | 2132.59 | ± | 11.81 | 8.08 | ± | 0.03 | -4.6 | 0.6 | 62.2 | -37.8 | <0.0001 |
| Vitamin C (mg) | 16.15 | ± | 0.08 | 60.78 | ± | 0.29 | -33.8 | 0.6 | 64.8 | -35.2 | <0.0001 |
| Vitamin B_6_ (mg) | 5.46 | ± | 0.02 | 0.65 | ± | 0.00 | -0.8 | 0.5 | 45.7 | -54.3 | <0.0001 |
| Folate (μg) | 7.08 | ± | 0.03 | 211.55 | ± | 0.72 | 17.1 | 1.1 | 113.9 | 13.9 | <0.0001 |
| Cholesterol (mg) | 126.98 | ± | 0.74 | 108.65 | ± | 0.62 | -18.3 | 0.9 | 88.2 | -11.8 | <0.0001 |

^1^ mean± standard error, ^2^ Paired t test
