# Supplementary material for: Nutrition survey methods and food composition database update of the Korean Genome and Epidemiology Study
Source: Epidemiol Health. 2024 Apr 2;46:e2024042. doi: 10.4178/epih.e2024042 (PMC11417449; doi:10.4178/epih.e2024042)
Supplement: Supplementary Material 3. — Coverage status of newly expanded nutrients [file epih-46-e2024042-Supplementary-3.docx]

**Supplementary Material 3.** Coverage status of newly expanded nutrients

| Nutrients | No of food | Coverage of 423 foods |
| --- | --- | --- |
| Total sugars (g) | 381 | 90.1 |
| Water-soluble dietary fiber (g) | 248 | 58.6 |
| Water-insoluble dietary fiber (g) | 248 | 58.6 |
| Vitamin D (μg) | 273 | 64.5 |
| Vitamin B_12_ (μg) | 421 | 99.5 |
| Magnesium (mg) | 275 | 65.0 |
| Iodine (μg) | 252 | 59.6 |
| Selenium (μg) | 268 | 63.4 |
| Copper (mg) | 275 | 65.0 |
| Histidine (mg) | 260 | 61.5 |
| Leucine (mg) | 260 | 61.5 |
| Isoleucine (mg) | 260 | 61.5 |
| Lysine (mg) | 260 | 61.5 |
| Valine (mg) | 260 | 61.5 |
| Phenylalanine (mg) | 260 | 61.5 |
| Threonine (mg) | 260 | 61.5 |
| Tryptophan (mg) | 256 | 60.5 |
| Methionine (mg) | 260 | 61.5 |
| Saturated fatty acid (g) | 412 | 97.4 |
| Monounsaturated fatty acid (g) | 410 | 96.9 |
| Polyunsaturated fatty acid (g) | 410 | 96.9 |
| Omega-3 fatty acid (g) | 405 | 95.7 |
| Omega-6 fatty acid (g) | 405 | 95.7 |
| Capric acid (mg) | 384 | 90.8 |
| Lauric acid (mg) | 385 | 91.0 |
| Myristic acid (mg) | 385 | 91.0 |
| Palmitic acid (mg) | 385 | 91.0 |
| Stearic acid (mg) | 385 | 91.0 |
| Arachidic acid (mg) | 383 | 90.5 |
| Behenic acid (mg) | 382 | 90.3 |
| Lignoceric acid (mg) | 380 | 89.8 |
| Myristoleic acid (mg) | 382 | 90.3 |
| Palmitoleic acid (mg) | 384 | 90.8 |
| Oleic acid (mg) | 378 | 89.4 |
| Gadoleic acid (mg) | 384 | 90.8 |
| Erucic acid (mg) | 384 | 90.8 |
| Nervonic acid (mg) | 380 | 89.8 |
| (Continued to the next page) | | |
| **Supplementary Material 3.** Continued | | |
| Nutrients | No of food | Coverage of 423 foods |
| Linoleic acid (mg) | 382 | 90.3 |
| α-linolenic acid (mg) | 383 | 90.5 |
| γ-linolenic acid (mg) | 382 | 90.3 |
| Stearidonic acid (mg) | 352 | 83.2 |
| Eicosadienic acid (mg) | 382 | 90.3 |
| Eicosatrienoic acid (mg) | 380 | 89.8 |
| Arachidonic acid (mg) | 380 | 89.8 |
| Eicosapentaenoic acid (mg) | 384 | 90.8 |
| Docosadienoic acid (mg) | 380 | 89.8 |
| Docosapentaenoic acid (mg) | 384 | 90.8 |
| Docosahexaenoic acid (mg) | 384 | 90.8 |
